# Dataset for human sensitivity to chemicals during development of motor function

**DOI:** 10.1016/j.dib.2015.12.036

**Published:** 2016-01-02

**Authors:** Susan Z. Ingber, Hana R. Pohl

**Affiliations:** Agency for Toxic Substances and Disease Registry, 1600 Clifton Rd, Mailstop F-57, Atlanta, GA 30333, United States

**Keywords:** Windows of sensitivity, Motor function, Developmental effects, Human exposure effects, *in utero* exposure effects

## Abstract

The authors reviewed human data related to motor development following exposure to a subset of chemicals thoroughly reviewed in Agency for Toxic Substances and Disease Registry (ATSDR) Toxicological Profiles and Addenda. The resulting dataset includes the following variables and confounders: chemical name, exposure route, exposure duration and frequency, study design, cohort name and/or geographic location, sex of cohort subjects, NOAEL, and LOAEL. This data summary can help validate motor development outcomes observed in animal exposure studies; it can also aid in determining whether these outcomes and corresponding exposure windows are relevant to humans.

**Specifications table**

**Table:** 

Subject area	*Biology*
More specific subject area	*Epidemiology, Toxicology, Environmental Health*
Type of data	*Table*
How data was acquired	*Literature search*
Data format	*Filtered*
Experimental factors	*N/A (Literature review)*
Experimental features	*N/A*
Data source location	*N/A*
Data accessibility	*Data are with this article*

**Value of the data**

• The human epidemiological data provide researchers with a condensed reference of chemicals for which motor development effects have been observed and at which doses and exposure durations.
• They can be compared to animal data to help assess relevance to humans.
• They provide a list of chemicals for which researchers can design much-needed acute duration studies on motor development effects known to occur in humans.

## 1 Data

Table 1 presents motor development outcome and study design data extracted from epidemiological studies of chemicals reviewed in ATSDR’s Toxicological Profiles and Addenda. The data include: No Observed Adverse Effect Levels (NOAELs) and/or Lowest Observed Adverse Effect levels (LOAELs), exposure routes and durations assessed, cohort name and demographics [geographic location, sex(es)], and study design type. Agency for Toxic Substances and Disease Registry Toxicological Profiles (http://www.atsdr.cdc.gov/toxprofiles/index.asp), NCBI’s PubMed (http://www.ncbi.nlm.nih.gov/pubmed).

## 2 Experimental design, materials and methods

As detailed in [26], the primary search utilized ATSDR’s Toxicological Profiles (*n*=173 chemicals) and Addenda (*n*=41 chemicals). We read through the profiles and addenda in search of data pertaining to motor function development in association with chemical exposure. In order to find human studies corroborating or challenging the results of the toxicological profile and addenda animal studies as well as assess additional chemicals known to affect motor development, further searches were done on PubMed using common text words and MeSH terms related to motor function development:

((((((((motor[All Fields] AND ("physiology"[Subheading] OR "physiology"[All Fields] OR "function"[All Fields] OR "physiology"[MeSH Terms] OR "function"[All Fields]))) OR (sensorimotor[All Fields] AND ("physiology"[Subheading] OR "physiology"[All Fields] OR "function"[All Fields] OR "physiology"[MeSH Terms] OR "function"[All Fields]))) OR (visual[All Fields] AND motor[All Fields])) OR graphomotor) OR gross motor function) OR (fine AND motor AND function))) AND ("developmental disabilities"[MeSH Terms] OR ("developmental"[All Fields] AND "disabilities"[All Fields]) OR "developmental disabilities"[All Fields]) and different chemicals.

Studies were not limited by date of publication, species, chemical, or study design; however, studies were limited to the English language. The following data from each study were extracted, when available: chemical name, exposure route, exposure duration and frequency, study design, cohort name and/or geographic location, sex of cohort subjects, NOAEL, and LOAEL.

## Figures and Tables

**Table 1:** Summary of human NOAEL & LOAEL data for motor development outcomes.

Chemical	Sex of offspring	Cohort (location)	Study design	Exposure duration/frequency	NOAEL (mg/kg/d)	LOAEL (mg/kg/d)	Reference

**Motor activity**							
Ethanol	M & F	Birth Cohort (Grady Memorial Hospital, Atlanta, Georgia)	Prospective cohort	Gestation (entirety or first two trimesters)	Not established	12 oz/week×entire gestation period, 14 oz/week×first and second trimesters (reduced motor maturity, increased activity)	[13]
DDE	M & F	Birth Cohort (Flix, Spain)	Prospective cohort	Gestation	Not established	Delay in psychomotor development observed at 13 months age; decreased locomotor performance on the Griffiths Mental Development Scales	[34]
ΣPBDEs	M & F	Menorca birth cohort/INMA (INfancia y Medio Ambiente [Environment and Childhood]) project (Spain)	Prospective cohort	Gestation (cord blood) and postnatal (4 years age)	2.10(16.8) ng/mL (Median(max)) cord blood; 0.12(130.2) ng/mL serum at age 4 (hyperactivity via ADHD criteria of DSM-IV)		[23]

**Motor function**							
Arsenic	M & F	Maternal and Infant Nutritional Intervention at Matlab (MINIMat) study (Matlab, Bangladesh)	Community-based randomized trial	8 or 30 weeks gestation	81 (37–207) μg/L at 8 weeks of gestation; 84 μg/L (42–230 μg/L) at 30 weeks (PDI score)		[43]
Arsenic	M & F	Children from Health Effects of Arsenic Longitudinal Study (HEALS) (Araihazar, Bangladesh)	Cross-sectional	Post-natal (8–11 years age; drinking water levels; blood levels)	Not established	Mean 6.3 μg/L in blood (decreased motor function – including motor coordination – via Bruininks–Oseretsky test)	[33]
Carbon monoxide	M & F	Birth Cohort (San Marcos, Guatemala)	Prospective cohort	Gestation, postnatal inhalation	Exposure during the first 9 postnatal months (fine motor function via Reitan-Indiana Finger tapping test, WRAVMA Pegboard test, and Bender-Gestalt-II connect the dots motor test); mean 3.8 ppm (fine motor function as measured by WRAVMA and Bender Gestalt-II tests)	Mean 3.8 ppm, range 0.62–12.52 ppm (decreased fine motor speed via Reitan-Indiana Finger tapping test: −5.7 (−9.7, −1.7)) with increasing maternal third trimester exposure level at age 6–7 years	[16]
Chlordecone	M & F	Timoun mother– child cohort (Guadaloupe)	Prospective cohort	Gestation	Median (range) <0.24 (0.07–3.91) μg/L F (fine motor function via Ages and Stages Questionnaire (ASQ) score converted to IQ score); M & F (gross motor function)	0.24 μg/L M (impaired fine motor function via converted ASQ score)	[12]
Chlordecone	M & F	Timoun mother– child cohort (Guadaloupe)	Prospective cohort	Gestation+lactation (breast milk sample measured when infant reached 3 months age)	Median (range) 0.62 (0.2–4.2) μg/L M & F (fine and gross motor function (Ages and Stages Questionnaire (ASQ) score converted to IQ score))		[12]
Chlorodibenzofurans (CDFs)	M & F	Yu-Cheng rice oil contamination victims (Taiwan)	Field survey (retrospective)	Gestation	Not established	Decreased PDI score	[37]
Total CDDs/CDFs+PCBs	M & F	Duisburg Birth Cohort Study (Germany)	Prospective cohort	Gestation	Mean (range) 18.8 (4.0–51.2) pg/g blood lipids TEQs (motor development (PDI) assessed by the Bayley Scales of Infant Development (at ages 12 and 24 months))		[45]
DDE	M & F	Birth Cohort (Flix, Spain)	Prospective cohort	Gestation	Not established	Delay in psychomotor development observed at 13 months age; decreased locomotor performance on the Griffiths Mental Development Scales	[34]
DDT, DDE	M & F	Center for the Health Assessment of Mothers and Children of Salinas study (Salinas Valley, California)	Prospective cohort	Gestation	Not established	A decrease of ~2 points in the psychomotor developmental index score (PDI) with each 10-fold increase in maternal p,p′-DDT blood serum levels (geometric mean (95% CI): 22.0 (18.4–26.4) ng/g lipid) when the children reached 6 and 12 months of age (but not at 24 months) and maternal p,p′-DDE levels (geometric mean (95% CI): 1436.9 (1257.4–1642.1) ng/g lipid) at 6 months of age only)	[21]
DDT, DDE, DDD (except o,p′-isomers)	M & F	Perinatal Cohort (Morelos, Mexico)	Prospective cohort	Gestation (exposure at each trimester measured)	6.8 (2.8) ng/mL (GM (GSD)) 2nd and 7.8 (2.8) (GM(GSD)) 3rd trimester exposure (PDI score at 3, 6 and 12 months age)	6.4 (2.8) ng/mL (GM (GSD)) 1st trimester exposure (PDI score at 3, 6 and 12 months age)	[44]
DDT, DDE, DDD (except o,p′-isomers)	M & F	Groningen infant COMPARE (Comparison of Exposure-Effect Pathways to Improve the Assessment of Human Health Risks of Complex Environmental Mixtures of Organohalogens) study	Prospective cohort	Gestation (35 weeks)	Not established	Improved coordination (via Touwen’s neurologic examination)	[41]
Ethanol	M & F	Birth Cohort (Grady Memorial Hospital, Atlanta, Georgia)	Prospective cohort	Gestation (entirety or first two trimesters)	Not established	12 oz/week×entire gestation period, 14 oz/week×first and second trimesters (reduced motor maturity, increased activity)	[13]
Ethanol	M & F	Pregnant women seeking prenatal care (U.S.)	Prospective cohort	Gestation (exposure measured at 5 months gestation)	Not established	≥.5 oz per day in early pregnancy (approximately 1 drink per day) decreased fine motor skill performance on the Wisconsin Fine Motor Steadiness Battery at 4 years age;	[2]
Decreased gross motor performance (includes motor coordination) on a battery adapted from the Gross Motor Scale developed by Crippled Chidren’s Division of the University of Oregon Medical School
Heptachlor epoxide	M & F	Oahu high school students exposed during gestation (Oahu)	Retrospective	Gestation	Not established	Impaired motor planning at high school age	[1]
Lead	M & F	Cincinnati Lead Study, Birth Cohort (Cincinnati, Ohio)	Prospective cohort	Gestation (first trimester, gestation+10 days (neonatal), gestation through age 6 (current level))	8.4 μg/dL (prenatal) (fine motor function)	Mean 4.8 (neonatal) and 10.1 μg/dL (current level)(decreased fine motor function) (Bruininks–Oseretsky Test of Motor Proficiency (BOTMP));	[15]
10.1 μg/dL (current level) (decreased motor coordination as per bilateral coordination subtest of BOTMP at age 6)
Lead	M & F	Birth Cohort (Cleveland, OH)	Prospective cohort	Gestation (cord blood measured on day of delivery)	Not established	Mean 5.8 (range, 2.6–14.7) μg/dL cord blood (neurological soft signs in newborn infants)	[17], [19], [18]
Lead	M & F	Birth Cohort (Cleveland, OH)	Prospective cohort	Gestation (maternal blood level measured on day of delivery)	Not established	Mean 6.5 (range, 2.7–11.8) μg/dL pre-natal maternal blood (altered Psychomotor Developmental Index)	[18]
Lead	M & F	Middle- and upper-class children (Boston, Massachusetts)	Prospective cohort	10 years (from birth)	Mean 7 μg/dL PbB at 24 months (Psychomotor Developmental Index)		[3], [4], [5], [6], [7], [8], [9], [10], [11]
Manganese	M & F	Mexican children (Chiconcoac and Tolago, Mexico)	Cross-sectional	Gestation+post-natal (lifetime exposure, elementary school aged)	Median 12.6 μg/g in hair (Motor function via finger tapping (Halstead-Reitan battery), grooved pegboard, and Santa Ana (motor coordination) tests); Median 9.5 μg/Lin blood (motor function via groove pegboard and Santa Ana tests)	Median 9.5 μg/Lin blood (decreased motor function via finger-tapping test (on-dominant hand only))	[25]
Manganese	M & F	public school children (Province of Brescia, Italy)	Cross-sectional	Post-natal (11–14 years age; soil levels)	Not established	958 ppm in soil (impairment of motor coordination (Luria–Nebraska test), hand dexterity (Pursuit Aiming));	[30]
10.99 μg/L in blood (increased tremor intensity);
0.16 ppm in hair (increased tremor intensity)
Manganese	M & F	Children from Health Effects of Arsenic Longitudinal Study (HEALS) (Araihazar, Bangladesh)	Cross-sectional	Post-natal (8–11 years age; drinking water levels; blood levels)	Motor function – including motor coordination -- via Bruininks–Oseretsky test		[33]
Mercury (organic)	M	Seychelles Child Development Study (Republic of Seychelles)	Prospective cohort	*In utero* exposure	Female arithmetic mean 6.9 ppm (<3–12 ppm) (performance on the grooved pegboard time for non-dominant hand)	Male arithmetic mean 6.9 ppm (<3–12 ppm) (decreased performance on the grooved pegboard time for non-dominant hand)	[31]
Mercury (organic)	F	Seychelles Child Development Study (Republic of Seychelles)	Prospective cohort	*In utero*+post-natal exposure, 107 months	Mean 6.6 ppm (6 months age), 4.8 ppm (66 months age), 6.9 ppm (107 months age); Male (performance on the grooved pegboard time for non-dominant hand)	Female mean 6.6 ppm (6 months age), 4.8 ppm (66 months age), 6.9 ppm (107 months age) (decreased performance on grooved Pegboard with the non-dominant hand test)	[32]
Mercury (organic)	M & F	Birth Cohort (Poland)	Prospective cohort	Prenatal exposure (maternal fish consumption)	Geometric mean 0.52 μg/mL maternal blood (MDI & PDI scores)	Geometric mean 0.75 μg/L maternal blood (reduced MDI & PDI scores)	[27]
Mercury (organic)	M & F	Mother-child pairs exposed during Iraqi poisoning incident (Iraq)	Prospective cohort?	Prenatal exposure (maternal consumption, measured in hair sample)	Not established	0.0012 (delayed walking; abnormal motor scores (includes assessment of ataxia, abnormal reflexes, and athetoid movement))	[14]
Methyl parathion	M & F	Children exposed during illegal spraying (Mississippi and Ohio, USA)	Cross-Sectional?	Post-natal (6 years age and under when homes sprayed with MP)	Integration of motor skills based on Pediatric Environmental Neurobehavioral Test Battery (PENTB)		[42]
ΣPBDEs	M & F	Menorca birth cohort/INMA (INfancia y Medio Ambiente [Environment and Childhood]) project (Spain)	Prospective cohort	Gestation (cord blood) and postnatal (4 years age)	2.10(16.8) ng/mL (Median(max)) cord blood; 0.12(130.2) ng/mL serum at age 4 (McCarthy Scales of Children׳s Abilities motor function score)		[23]
ΣPBDEs	M & F	CHAMACOS cohort (Salinas, California)	Prospective cohort	Gestation (maternal blood at 26.7±2.6 weeks gestation or delivery) and postnatal (child serum at age 7 years)	Gestation and postnatal levels (Gross motor function via McCarthy Scales of Children׳s Abilities (MSCA)); postnatal levels (motor function via finger tap test, WRAVMA pegboard test, and MSCA gross motor test)	Gestational exposure (decreased motor function at ages 5 and 7) via WRAVMA pegboard test (non-dominant hand)	[20]
BDE 47	M & F	Groningen infant COMPARE (Comparison of Exposure-Effect Pathways to Improve the Assessment of Human Health Risks of Complex Environmental Mixtures of Organohalogens) study	Prospective cohort	Gestation (35 weeks)	Not established	Improved coordination (via Touwen’s neurologic examination), but the effect disappeared after correcting for SES, sex, and Home Observation for Measurement of the Environment (HOME) questionaire results	[41]
BDE 100	M & F	Groningen infant COMPARE (Comparison of Exposure–Effect Pathways to Improve the Assessment of Human Health Risks of Complex Environmental Mixtures of Organohalogens) study	Prospective cohort	Gestation (35 weeks)	Not established	Improved coordination (via Touwen’s neurologic examination), but the effect disappeared after correcting for SES, sex, and Home Observation for Measurement of the Environment (HOME) questionaire results	[41]
BDE 154	M & F	Groningen infant COMPARE (Comparison of Exposure–Effect Pathways to Improve the Assessment of Human Health Risks of Complex Environmental Mixtures of Organohalogens) study	Prospective cohort	Gestation (35 weeks)	Not established	Decreased fine manipulative abilities (via Touwen’s neurologic examination), but the effect disappeared after correcting for SES, sex, and Home Observation for Measurement of the Environment (HOME) questionaire results (*p*<0.1)	[41]
HBCDD	M & F	Groningen infant COMPARE (Comparison of Exposure–Effect Pathways to Improve the Assessment of Human Health Risks of Complex Environmental Mixtures of Organohalogens) study	Prospective cohort	Gestation (35 weeks)	Not established	Improved coordination (via Touwen’s neurologic examination)	[41]
Pentachlorophenol (PCP)	M & F	Groningen infant COMPARE (Comparison of Exposure–Effect Pathways to Improve the Assessment of Human Health Risks of Complex Environmental Mixtures of Organohalogens) study	Prospective cohort	Gestation (35 weeks)	Not established	Decreased coordination (via Touwen’s neurologic examination)	[41]
Perfluoroalkyls	M & F	Danish National Birth Cohort (Denmark)	Prospective cohort	Gestation (maternal plasma)	Fine and gross motor function at ages 6 and 18 months (neurological consult)		[22]
Polychlorinated Biphenyls (PCBs)	M & F	Birth Cohort (North Carolina)	Prospective cohort	Gestation	1.8 ppm breast milk (lipid based) (Postnatal exposure to PCBs was not associated with the PDI at 6 or 12 months)	Median 9.06 ppb maternal serum; median <4.27 ppb cord serum (At 6 months, the PDI was estimated to decrease 0.96 points for every increase of 1 ppm in PCBs. This would mean a drop of 2.6 points if a child moved from the 5th to the 95th percentile of PCB exposure. At 12 months, the drop was estimated at 1.34 points/ppm)	[24], [29], [36], [35], [38], [40], [39]
Polychlorinated Biphenyls (PCBs)	M & F	Dutch PCB/Dioxin Study (Rotterdam, Denmark)	Prospective cohort	Gestation	2.2 ppb maternal plasma, 0.45 ppb cord plasma (PDI at 7 and 18 months age)	2.2 ppb maternal plasma, 0.45 ppb cord plasma (decreased PDI at 3 months age)	[28]
Polychlorinated Biphenyls (PCBs)	M & F	Dutch PCB/Dioxin Study (Rotterdam, Denmark)	Prospective cohort	Gestation+2 weeks lactation	Not established	Median 0.75 ppb child plasma, 0.046 ppb TEQ (decreased PDI at 7 months among infants who were breastfed for longer periods and had higher TEQ scores were associated with postnatal total TEQ exposure)	[28]
